# The flavin mononucleotide cofactor in α-hydroxyacid oxidases exerts its electrophilic/nucleophilic duality in control of the substrate-oxidation level

**DOI:** 10.1107/S2059798319011938

**Published:** 2019-09-24

**Authors:** Syue-Yi Lyu, Kuan-Hung Lin, Hsien-Wei Yeh, Yi-Shan Li, Chun-Man Huang, Yung-Lin Wang, Hao-Wei Shih, Ning-Shian Hsu, Chang-Jer Wu, Tsung-Lin Li

**Affiliations:** a Genomics Research Center, Academia Sinica, Taipei 115, Taiwan; b Institute of Biochemistry and Molecular Biology, National Yang-Ming University, Taipei 112, Taiwan; c Department of Food Science, National Taiwan Ocean University, Keelung 202, Taiwan; d Biotechnology Center, National Chung Hsing University, Taichung City 402, Taiwan

**Keywords:** electrophilic/nucleophilic duality, α-hydroxyacid oxidases, flavin mononucleotide, oxidative decarboxylation, monooxygenase, *p*-hydroxymandelate oxidase

## Abstract

The Y128F single mutant of *p*-hydroxymandelate oxidase (Hmo) is capable of oxidizing mandelate to benzoate via a four-electron oxidative decarboxylation reaction. When benzoylformate (the product of the first two-electron oxidation) and hydrogen peroxide (an oxidant) were used as substrates the reaction did not proceed, suggesting that free hydrogen peroxide is not the committed oxidant in the second two-electron oxidation. How the flavin mononucleotide (FMN)-dependent four-electron oxidation reaction takes place remains elusive. Structural and biochemical explorations have shed new light on this issue. 15 high-resolution crystal structures of Hmo and its mutants liganded with or without a substrate reveal that oxidized FMN (FMN_ox_) possesses a previously unknown electrophilic/nucleophilic duality. In the Y128F mutant the active-site perturbation ensemble facilitates the polarization of FMN_ox_ to a nucleophilic ylide, which is in a position to act on an α-ketoacid, forming an N5-acyl-FMN_red_ dead-end adduct. In four-electron oxidation, an intramolecular disproportion­ation reaction via an N5-alkanol-FMN_red_ C′α carbanion intermediate may account for the ThDP/PLP/NADPH-independent oxidative decarboxylation reaction. A synthetic 5-deaza-FMN_ox_ cofactor in combination with an α-hydroxyamide or α-ketoamide biochemically and structurally supports the proposed mechanism.

## Abstract 2

Structural and enzymological explorations of *p*-hydroxy-mandelate oxidase and its mutants uncover an unprecedented electrophilic/nucleophilic duality for the flavin mononucleotide cofactor as well as an intramolecular disproportionation mechanism for an oxidative decarboxylation reaction.

## 1. Introduction   

**Figure 1:**
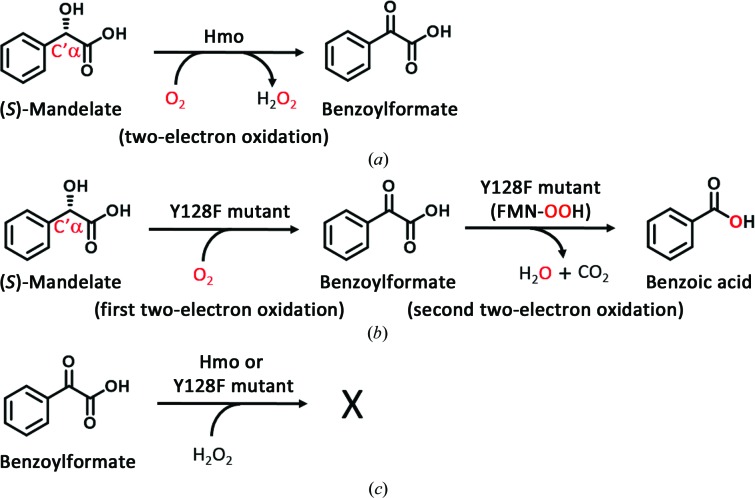
The oxidation reactions catalyzed by Hmo and its Y128F mutant. (*a*) Hmo catalyzes a two-electron oxidation reaction to form benzoylformate from (*S*)-mandelate. (*b*) The Y128F mutant catalyzes a four-electron oxidative decarboxylation reaction from (*S*)-mandelate to benzoylformate and benzoic acid without freeing H_2_O_2_ during the reaction. (*c*) When benzoylformate is used as the substrate, the decarboxylated product benzoic acid cannot be formed by Hmo or its Y128F mutant in the presence or absence of H_2_O_2_.

*p*-Hydroxymandelate oxidase (Hmo) is a flavin mononucleotide (FMN)-dependent enzyme that oxidizes mandelate to benzoylformate. Its Y128F single mutant unexpectedly shows a new reactivity and is able to oxidize mandelate to benzoate via benzoylformate, a four-electron oxidation reaction that is typically catalysed by a monooxygenase. However, when using benzoylformate in place of mandelate the reaction becomes stuck in the absence or the presence of hydrogen peroxide (H_2_O_2_; Yeh *et al.*, 2019 ▸; Fig. 1 ▸). To the best of our knowledge, this is the second example after lactate monooxygenase (LMO) of an enzyme that performs a ThDP/PLP/NADPH-independent oxidative decarboxylation reaction at the expense of one molecule of O_2_ with the concomitant production of CO_2_ and H_2_O (Ghisla & Massey, 1989 ▸). It has been hypothesized that the H_2_O_2_ generated at the active site of LMO acts on pyruvate to form acetate by H_2_O_2_-mediated oxidative decarboxylation because the dissociation of pyruvate is a slow step (Giegel *et al.*, 1990 ▸; Lopalco *et al.*, 2016 ▸). Aside from this non-ping-pong kinetic description, how H_2_O_2_ mediates the oxidative decarboxylation of an α-ketoacid at the molecular level remains elusive (Choong & Massey, 1980 ▸; Ghisla & Massey, 1977 ▸; Lockridge *et al.*, 1972 ▸; Walsh *et al.*, 1973 ▸). It has been noted that the electron reactivity of FMN in flavin-dependent enzymes is the main factor governing the implementation of a given type of reaction by the dioxygen, substrates and enzyme–cofactor system. C4α and N5 of the isoalloxazine ring of FMN are the most reactive centers, electrophilically or nucleophilically interacting with a substrate through a covalent linkage or spatiotemporally conveying electrons during redox reactions (Walsh & Wencewicz, 2013 ▸). In a previous study (Yeh *et al.*, 2019 ▸), benzoylformate was found to be able to adopt a *pro-R* orientation with reference to the *si* face of the iso­alloxazine ring. This structural re­orientation aligns the nucleophilic N5 or C4α-OOH of reduced FMN (FMN_red_) with an appropriate trajectory to the electrophilic α-keto carbon of the α-ketoacid, a reaction reminiscent of the suicide inhibition of monoamine oxidase by mofegiline by forming an inactive N5 adduct or the formation of indole-3-acetate in auxin biosynthesis by the NADPH flavin-dependent monooxygenase YUC via a C4α-OOH decarboxylation-assisted mechanism (Wu *et al.*, 2005 ▸; Stepanova *et al.*, 2011 ▸; Milczek *et al.*, 2008 ▸; Dai *et al.*, 2013 ▸). In this report, both biochemical and structural measures were exploited in an attempt to address how a single O atom dictates the oxidation level in the reactions catalyzed by wild-type Hmo and its Y128F mutant.

## 2. Materials and methods   

### 2.1. Cloning and protein purification   

The *hmo* gene (*orf22*) was amplified from *Amycolatopsis orientalis* genomic DNA by polymerase chain reaction and was subcloned into the expression vector pET-28a(+) to generate the protein with an N-terminal His_6_ tag. The expression plasmid was transformed into *Escherichia coli* BL21(DE3) cells by electroporation and the cells were grown on LB agar plates containing 50 µg ml^−1^ kanamycin for 16 h at 37°C. A single colony was grown overnight in 5 ml LB medium containing 50 µg ml^−1^ kanamycin at 37°C. The cell culture was used to inoculate 1 l LB medium containing 50 µg l^−1^ kanamycin. For protein expression and purification, the transformed *E. coli* cells were cultured, induced with 200 µl 1.0 *M* IPTG at an OD_600_ of 0.6 and grown for a further 24 h at 16°C. The cells were harvested by centrifugation, resuspended in 20 ml binding buffer (20 m*M* HEPES pH 7.5, 500 m*M* NaCl, 10 m*M* imidazole, 10% glycerol) and lysed using a microfluidizer or by sonication. The supernatant of the lysate after centrifugation at 16 000 rev min^−1^ for 30 min was applied onto an Ni^2+^–NTA resin column. The bound protein was sequentially washed with 10 ml binding buffer (50 m*M* HEPES pH 8, 200 m*M* NaCl, 20 m*M* imidazole) and 10 ml wash buffer (50 m*M* HEPES pH 8, 200 m*M* NaCl, 80 m*M* imidazole) before elution with 10 ml elution buffer (20 m*M* HEPES pH 8, 500 m*M* NaCl, 250 m*M* imidazole). Gel filtration was performed using an ÄKTA FPLC system equipped with a HiLoad 16/60 Superdex 200 column (Amersham Bioscience) under isocratic conditions (20 m*M* HEPES pH 8, 100 m*M* NaCl).

### 2.2. Crystallization and data collection   

The purified proteins were crystallized using the hanging-drop vapor-diffusion method. Hmo and its Y128F, Y128C and R163L mutants were concentrated to 7 mg ml^−1^ in 50 m*M* HEPES pH 8.0 buffer solution and crystallized using a solution consisting of 35% Tascimate, 0.1 *M* bis-Tris propane pH 7.0 in a 50:50 volume ratio. Crystals appeared within five days in VDX48 plates (Hampton Research) with sealant at 20°C. For Hmo and its mutants in complex with (*S*)-mandelate (SMA), benzoylformate (BF), benzoic acid (BA), phenyl­pyruvate (PPY), oxaloacetate (OAA) or mandelamide (MAAD), each crystal was soaked with 10–30 m*M* of the designated ligand dissolved in the mother solution for between 10 min and 24 h before data collection. All crystals were transferred to a solution containing cryoprotectants and flash-cooled in liquid nitrogen prior to data collection. The cryoprotectant-containing solution for Hmo and the Y128F mutant consisted of 20%(*w*/*v*) glycerol and 35% Tascimate. X-ray diffraction data were recorded at an operating temperature of 100 K using an ADSC Quantum 315 or an MX300HE CCD detector on beamlines 13B1, 13C1, 15A1 or 05A at the National Synchrotron Radiation Research Center, Taiwan or beamline 44XU at SPring-8, Japan. All of the crystals belonged to the same space group: *I*422.

### 2.3. Structure determination and refinement   

Data were indexed and scaled using the *HKL*-2000 package (Otwinowski & Minor, 1997 ▸). The crystal structures were determined by the molecular-replacement (MR) method using *Phaser-MR* from the *CCP*4 suite (McCoy *et al.*, 2007 ▸; Winn *et al.*, 2011 ▸). The crystal structure of hydroxyacid oxidase (PDB entry 3sgz; Chen *et al.*, 2012 ▸) was used as the search model for solving the initial phase. The polypeptide structures were built and refined using *REFMAC* (Murshudov *et al.*, 2011 ▸). Subsequent iterative cycles of model building and refinement were performed using *Coot* (Emsley *et al.*, 2010 ▸) and *PHENIX* (Afonine *et al.*, 2012 ▸). All non-H atoms were refined with anisotropic displacement parameters. Both protein structures and electron-density maps were generated using *PyMOL* (DeLano, 2002 ▸). Detailed refinement statistics are presented in Table 1 ▸.

### 2.4. Site-directed mutagenesis   

The site-directed Hmo point mutants Y128C, Y128F and R163L were generated using QuikChange (Stratagene). The primers for mutant preparation are listed in Supplementary Table S1. The mutations were confirmed by DNA sequencing. Each mutated protein was purified using the same protocol as used for wild-type Hmo.

### 2.5. Chemoenzymatic synthesis of 5-deazaflavin mononucleotide   

To obtain 5-deazaflavin mononucleotide, the commercially available compounds 3,4-dimethylaniline, sodium cyanoboro­hydride and d-ribose were used as starting materials, following the synthetic procedure described in Supplementary Scheme S1. Chemically synthesized deazariboflavin was purified using column chromatography, characterized by mass spectrometry or NMR and converted to catalytically active 5-deaza-FMN by riboflavin kinase. The gene coding for riboflavin kinase was amplified from the genomic DNA of *E. coli* K12 and was subcloned into a pET-28a vector to carry an N-terminal His_6_ tag. The constructed plasmid was then transformed into *E. coli* BL21(DE3) cells for overexpression. The overexpressed protein was purified using Ni^2+^–NTA affinity chromatography and concentrated to 15 mg ml^−1^. The reaction was initiated by the addition of 2 mg ml^−1^ riboflavin kinase to a reaction solution consisting of HEPES pH 7.5 with 5 m*M* ATP, 5 m*M* MgSO_4_ and 10 m*M* 5-deazariboflavin overnight. The sample solution was purified by HPLC and confirmed by mass spectrometry.

### 2.6. Preparation of 5-deaza-FMN-containing Hmo and its Y128F mutant   

The deflavination and reconstitution of Hmo and its Y128F mutant were performed on an Ni^2+^–NTA resin column by circulating a solution of HEPES buffer containing 10 m*M* deazariboflavin (Hefti *et al.*, 2003 ▸). The Ni^2+^–NTA resin column was loaded and saturated with a solution of the target protein at a flow rate of 0.2 ml min^−1^ using a peristaltic pump, which was installed between the sample reservoir and the column, to ensure maximal binding. Ten volumes of denaturing buffer (50 m*M* HEPES, 2 *M* potassium bromide, 2 *M* urea pH 8.0) were subsequently used to unfold Hmo or its Y128F mutant, allowing removal of the FMN prosthetic cofactor from Hmo or its Y128F mutant. On-column replacement with 5-deaza-FMN was completed by circulating a buffer solution consisting of 50 m*M* HEPES pH 8.0, 10 m*M* 5-deaza-FMN at 0.2 ml min^−1^ overnight. Hmo or its Y128F mutant containing 5-deaza-FMN was eluted using 10 ml elution buffer (20 m*M* HEPES pH 8, 200 m*M* NaCl, 250 m*M* imidazole). An analytical gel-filtration experiment using a Superdex 200 16/20 column confirmed that 5-deaza-FMN-containing Hmo (5-deaza-Hmo) and its Y128F mutant have an unchanged tetrameric state after the refolding process. The purified enzymes were concentrated to 10 mg ml^−1^ for enzymatic assays and protein crystallization.

### 2.7. Enzymatic assays using 5-deaza-Hmo and its Y128F mutant   

A typical enzymatic reaction (10 µg 5-deaza-Hmo or its Y128F mutant) was carried out in 200 µl buffer solution (20 m*M* HEPES, 100 m*M* NaCl pH 7.5, 5 m*M* *S*-mandelate, benzoylformate or benzoic acid) at 25°C for 2 h. Reactions were quenched using 6 *N* HCl and subjected to HPLC-MS analysis (using an Agilent 1260 Infinity Quaternary LC module connected to a Thermo-Finnigan LTQ-XL). Analytes were separated using a reverse-phase C_18_ column (4.6 × 250 mm, 5 µm, C_18_ Prodigy, Phenomenex) at a flow rate of 1 ml min^−1^ in a mobile system programmed as a linear gradient from 2% to 40% solvent *B* against solvent *A* over 25 min followed by 98% solvent *B* for a further 8 min (solvent *A*, water with 1% formic acid; solvent *B*, acetonitrile with 1% formic acid). The data were recorded using a UV detector with the wavelength set to 254/280 nm and the mass spectrometer in positive mode.

### 2.8. UV–Vis absorption measurements of reactions of Hmo and its Y128F mutant   

Ultraviolet and visible absorption spectra were recorded using a Beckman spectrophotometer (DU-800). In aqueous solution, freshly prepared Hmo or its Y128F mutant (0.125 m*M* in 0.05 *M* HEPES, 0.1 *M* NaCl pH 7.5) were mixed with substrates (2.5–5 m*M*) and incubated in a quartz cuvette at ambient temperature for 2 h. The absorption spectra of the reactions were recorded from 300 to 600 nm. For the redissolved crystals/crystalloids, more than 100 crystals of Hmo or its Y128F mutant (after soaking with substrates at 5 m*M* for 2 h) were picked from hanging-drop crystallization plates and redissolved in the mother liquor in a UV quartz cuvette before spectral scanning.

## 3. Results and discussion   

### 3.1. Inhibition of α-ketoacids   

**Figure 2:**
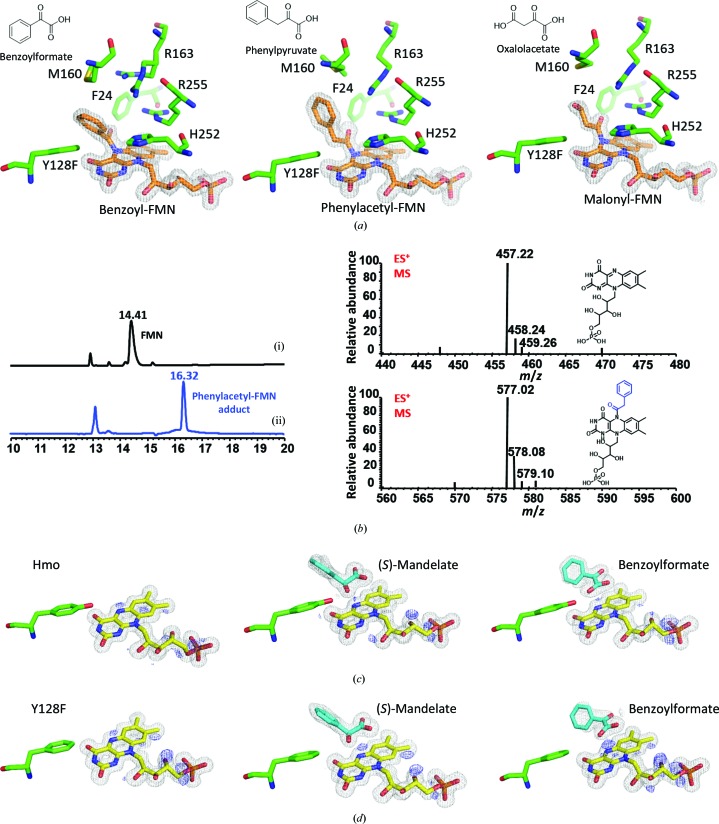
Crystal structures of acyl-FMN_red_ adducts and inhibition mechanism by α-ketoacids. (*a*) Structures of acyl-FMN_red_ adducts in crystals of the Y128F mutant soaked with benzoylformate (left), phenylpyruvate (center) or oxaloacetate (right). The flavin adducts all are at the *si*-face of the isoalloxazine ring. (*b*) LC traces and mass spectra of FMN (i) and phenylacetyl-FMN_red_ (ii). (*c*, *d*) Weighted 2*F*_o_ − *F*_c_ electron-density maps (gray) and unbiased *F*_o_ − *F*_c_ difference OMIT electron-density maps (blue) for FMN in Hmo (*c*) and the Y128F mutant (*d*) without (left) or with a ligand (*S*-mandelate, center; benzoylformate, right), where the extent of polarization is justified by OMIT electron density (the wild type or Y128F mutant and the absence or presence of a ligand seem to be key factors). The 2*F*_o_ − *F*_c_ electron-density map is contoured at 2σ; the unbiased *F*_o_ − *F*_c_ OMIT difference electron-density map is contoured at 4σ. Free ligands, FMN, FMN adducts and active-site residues are colored cyan, yellow, orange and green, respectively. See Supplementary Figs. S3(*a*), S3(*b*) and S2(*c*) for stereoviews and *F*_o_ − *F*_c_ difference electron-density maps.

To investigate the mechanism of the four-electron oxidative decarboxylation reaction catalyzed by the Hmo single mutant Y128F, we first solved crystal structures of the Y128F mutant in complex with different ligands such as (*S*)-mandelate, (*S*)-2-phenylpropionate, benzoylformate, benzaldehyde and benzoate. The ternary complexes of Hmo and its Y128F mutant were then compared, whereupon it was observed that the superpositioned complexes showed no apparent structural variations between Hmo and the Y128F mutant (r.m.s.d. of <0.1 Å; Supplementary Fig. S2) in terms of chemical conformations and spatial positions of both the proteins and ligands. When the crystals were soaked with benzoylformate, the formation of an N5-benzoyl-FMN adduct with an N5–C′α linkage was observed [Fig. 2 ▸(*a*)]. MS analysis confirmed that the benzoyl moiety was covalently linked to FMN [Fig. 2 ▸(*b*)]. When the crystals were soaked with benzaldehyde, the formation of a covalent adduct was not observed. This outcome suggests that the α-ketoacid moiety is a prerequisite for the formation of the covalent adduct, in which the decarboxylation of the terminal carboxyl group of the α-ketoacid is likely to take place after the formation of the N5–C′α linkage. Moreover, when the crystals of the Y128F mutant were soaked with (*S*)-3-phenyllactate, phenylpyruvate or phenylacetate [Fig. 2 ▸(*a*)], we observed that (*S*)-3-phenyllactate was oxidized to phenylacetate (a four-electron oxidative decarboxylation), phenylpyruvate ended up as an N5-phenylacetyl-FMN adduct and phenylacetate stayed as it was. When the crystals were soaked with oxaloacetate, an N5-malonyl-FMN adduct was found [Fig. 2 ▸(*a*)], leading overall to the conclusion that the formation of the covalent N5 adducts is α-ketoacid-dependent.

**Figure 3:**
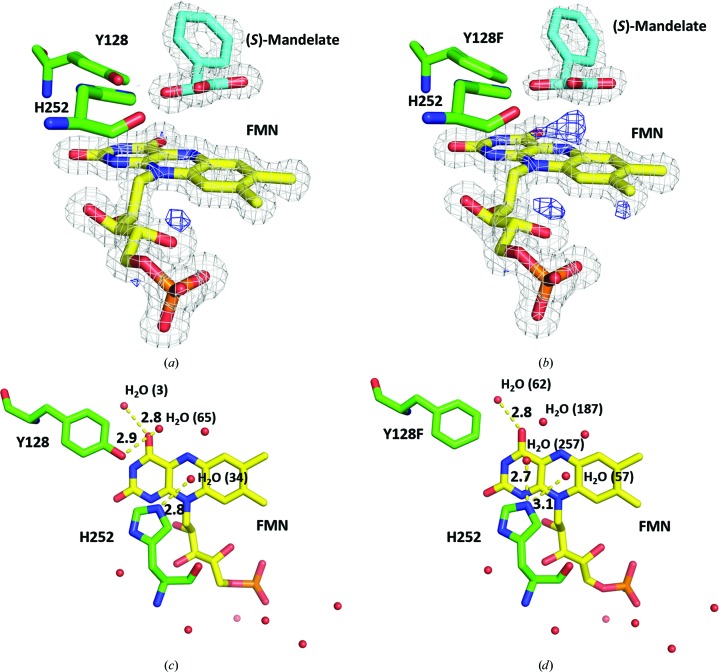

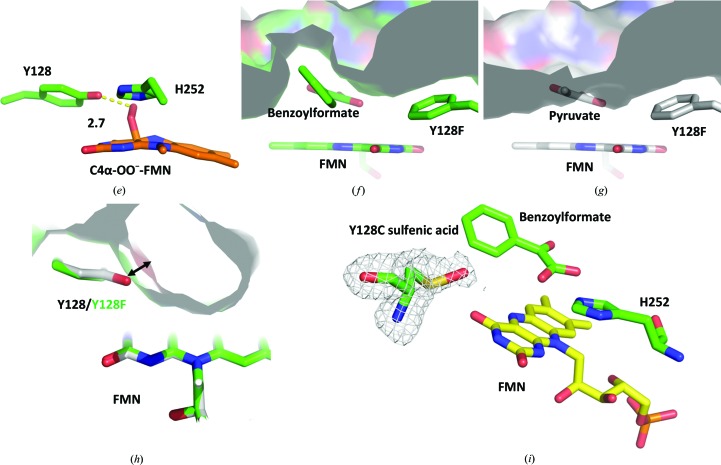
The effect of 4-OH of Tyr128 on polarization, hydrogen-bond networking and reactivity. (*a*, *b*) A close-up view of the wedge-shaped electron density on top of C4α=N5 of FMN_ox_ shown by unbiased difference electron-density maps (blue, positive electron density) contoured at 4σ in the wild type (*a*) and the Y128F mutant (*b*), suggesting that the electrons in the π-orbital of C4α=N5 are polarized from C4α to N5 (to form a C4α^+^–N5^−^ ylide), with this being more significant in the Y128F mutant than in the wild type. (*c*, *d*) The point mutation Y128F disturbs the active-site hydrogen-bonding network between water, FMN and the catalytic dyad [the Y128F mutant loses the hydrogen bond between Tyr128 and H_2_O (187) but gains a new hydrogen bond between His252 and H_2_O (257)]. (*e*) Hydrogen peroxide was modeled at C4α, where the distance between Tyr128 and the terminal O atom of Cα-OOH is 2.7 Å. (*f*, *g*) The phenyl ring of benzoylformate, which is bulkier and takes up space, limits the access of dioxygen to the reaction center, while the methyl group of pyruvate, which is smaller and takes up less space, allows the access of dioxygen to the reaction center [the phenyl ring that bulges out at the substrate entrance in (*f*) prohibits exposure of FMN_red_ to the bulk solvent, as opposed to the methyl group in (*g*) which allows exposure of FMN_red_ to the bulk solvent]. Therefore, the size of the substrates is another factor in leverage of the oxidation cascade. (*h*) Aside from the active-site perturbation effect, the absence of the *p*-­OH group also introduces some space allowing access of O_2_ to the C4α redox-active center. (*i*) The sulfhydryl group (SH) of the Y128C mutant has been oxidized to a sulfenyl group (S-OH), as it is vulnerable to ROS generated in the active site. Free ligands, FMN and active-site residues are colored cyan, yellow and green, respectively.

In most flavin-dependent oxidoreductases the FMN_ox_ cofactor acts as an electron sink (electrophile) that accepts electrons or hydrides conveyed from a substrate or NADH/NADPH in the reductive half-reaction. The nitroalkane oxidase from the fungus *Fusarium oxysporum* and the alkyldihydroxyacetone phosphate synthase in human fibroblasts are two rare cases in which a carbanion is generated at the active site prior to addition to N5 of the flavin cofactor as a covalent adduct (Razeto *et al.*, 2007 ▸; Heroux *et al.*, 2009 ▸). However, a question arises in the context of how two electrophiles (FMN_ox_ and α-ketoacid) are covalently associated in the Y128F mutant. One likelihood is that the α-ketoacid undergoes decarboxylation in the first instance to form a localized C′α carbanion that then acts on FMN_ox_, but an α-ketoacid that spontaneously undergoes decarboxylation is chemically untenable. The second possibility is that the *sp* ^2^ N5 atom of FMN_ox_ acts as a nucleophile, but this scenario likewise contradicts the current understanding: FMN_ox_ is a strong electrophile that accepts electrons. Nevertheless, an extra chunk of electron density at the top of N5 of FMN_ox_ was observed in unbiased difference electron-density maps, and this electron density was denser in the Y128F mutant than in the wild type [Figs. 2 ▸(*c*) and 2 ▸(*d*)]. This additional electron density suggests that the C4α=N5 double bond in FMN_ox_ is polarized to a C4α^+^–N5^−^ ylide (a tertiary carbocation and a tetrahedral *sp* ^3^ amine anion), as manifested by uneven wedge-shaped electron density for the π-bond [Figs. 2 ▸(*c*), 2 ▸(*d*), 3 ▸(*a*) and 3 ▸(*b*)]. The extent of polarization appears to be a function of an active-site perturbation ensemble (*e.g.* the point mutation), reflecting cooperative interplay of the hydrogen-bond network between water, FMN and active-site residues as well as ligands [Figs. 3 ▸(*c*) and 3 ▸(*d*)]. The formation of the adduct is thereby proposed to take place as follows: the *sp* ^3^ N5 atom of the polarized FMN_ox_ attacks the carbonyl C atom of the α-ketoacid to form a covalent C′α–N5 adduct, whereupon decarboxylation takes place, resulting in a localized C′α carbanion. The lone pair of the C′α carbanion subsequently hybridizes with the π orbital of N5 to reinstate the neutrality of C4α. Upon collapse of the C′α oxyanion, N5-acyl-FMN_red_ results via a series of bond rearrangements. This species has a hydroquinone-like structure, with the acyl moiety protruding out of the plane defined by the isoalloxazine ring of FMN_ox_ [Fig. 2 ▸(*a*)].

**Figure 4:**
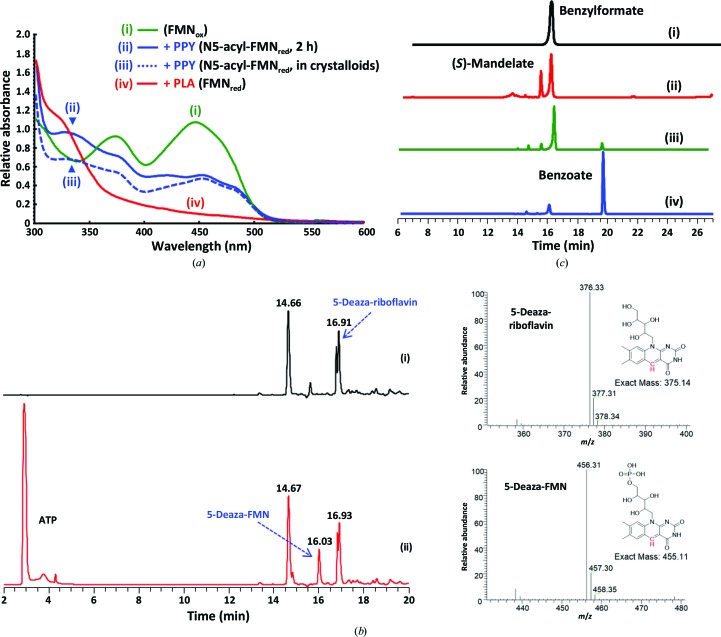

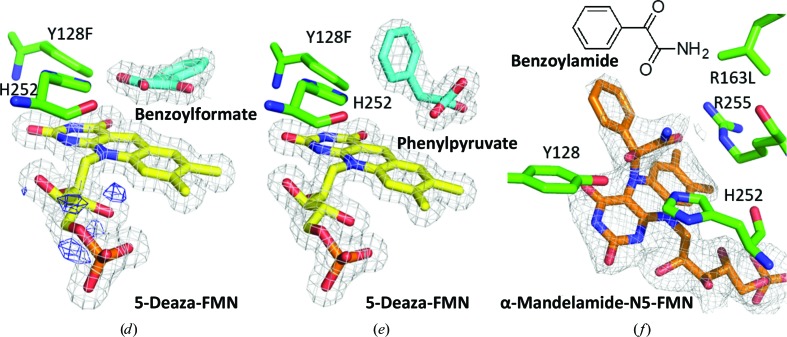
Spectra, synthesis, reaction and structure of 5-deaza-FMN. (*a*) UV–Vis spectra of FMN in Hmo and its Y128F mutant: (i) FMN_ox_ in Hmo (370/450 nm, green line), (ii) acyl-FMN_red_ in the Y128F mutant [340 nm, blue line; addition of phenylpyruvate (PPY) for 2 h], (iii) acylated FMN in redissolved Y128F mutant crystals/crystalloids soaked with PPY (acyl-FMN_red_, 340 nm, dotted blue line), (iv) FMN_red_ in Hmo [a shoulder at 330 nm, red; addition of phenyllactate (PLA)]. (*b*) (i) Chemical synthesis and LC purification of 5-deazariboflavin (the inset shows the mass spectrum of 5-deazariboflavin), (ii) enzymatic synthesis and LC purification of 5-deaza-FMN (the inset shows the mass spectrum of 5-deaza-FMN). (*c*) Enzymatic reactions of Hmo and its Y128F mutant harboring 5-deaza-FMN: (i) enzymatic reaction with Hmo harboring 5-deaza-FMN in the presence of *S*-mandelate, (ii) enzymatic reaction with the Y128F mutant harboring 5-deaza-FMN in the presence of *S*-mandelate, (iii) control reaction of wild-type Hmo in the presence of *S*-mandelate, (iv) control reaction of the Y128F mutant in the presence of *S*-mandelate. (*d*, *e*) Crystal structures of the Y128F mutant harboring 5-deaza-FMN soaked with *S*-mandelate (*d*) or phenyllactate (*e*), which have been transformed into benzoylformate or phenylpyruvate, respectively. Unlike FMN in the wild type or the Y128F mutant, no electron density emerges at the top of C5 or between C′α and C5. (*f*) The structure of an α-mandelamide–N5-FMN_red_ adduct in the crystal of the R163L mutant soaked with nondecarboxylable α-mandelamide (the chemical structure is shown). The flavin adduct is on the *si*-face of the isoalloxazine ring. The 2*F*_o_ − *F*_c_ electron-density map is contoured at 2σ. The unbiased *F*_o_ − *F*_c_ difference electron-density map is contoured at 4σ in positive electron density. Free ligands, FMN, FMN adducts and active-site residues are colored cyan, yellow, orange and green, respectively. See Supplementary Figs. S2(*j*)–S2(*m*) for stereoviews and 2*F*_o_ − *F*_c_ difference electron-density maps.

The UV–Vis spectrum of the Y128F mutant protein solution exhibits a typical FMN_ox_ absorbance profile [two absorbances at 370 and 450 nm; Fig. 4 ▸(*a*), i]. FMN_ox_ turned colorless when phenylpyruvate was added to the solution, suggesting the formation of an acyl-FMN species [Fig. 4 ▸(*a*), ii]. The spectrum is dissimilar to that observed when phenyllactate was added [in which the two typical absorbances at 370 and 450 nm disappeared, suggesting the reduction of FMN_ox_ to FMN_red_; Fig. 4 ▸(*a*), iv] by a small hump at 340 nm [Fig. 4 ▸(*a*), ii]. The redissolved solution of Y128F mutant crystals/crystalloids that had been pre-soaked with phenylpyruvate also turned colorless, with a profile similar to that in solution [with a smaller hump at 340 nm; Fig. 4 ▸(*a*), iii] (Sucharitakul *et al.*, 2007 ▸; Thotsaporn *et al.*, 2011 ▸). While O_2_ is a small hydrophobic molecule that freely diffuses through spaces and tunnels in protein matrices (Baron, McCammon *et al.*, 2009 ▸; Baron, Riley *et al.*, 2009 ▸), Hmo may have evolved a discrete channel or pockets that temporarily limit the access of O_2_ to C4α of N5-acylated isoalloxazine. The metastable N5-alkyl-FMN_red_ in aqueous solution is thus attributable to dysfunction of the charge-transfer cage in the absence of Hmo or its Y128F mutant. The inhibited Hmo and mutants identified here differ from the conventional inactivation of FMN_ox_, which requires chemically activated agents (Walsh, 1980 ▸, 1984 ▸).

### 3.2. 5-Deaza-FMN in oxidation   

A photoreduction mechanism has been proposed for the LMO-mediated oxidative decarboxylation reaction (Ghisla & Massey, 1977 ▸, 1989 ▸; Ghisla *et al.*, 1979 ▸), in which the reactants need to be activated by photosensitization. In the present case (Hmo and its Y128F mutant), the C4α^+^−N5^−^ ylide of FMN_ox_ appears to be the key factor. To validate this proposition, we chemoenzymatically synthesized 1 g of the FMN analog 3,10-dimethyl-5-deaza-isoalloxazine ribitol phosphate (5-deaza-FMN_ox_) following previously reported methods [Fig. 4 ▸(*b*); the modified method is described in the supporting information] (Carlson & Kiessling, 2004 ▸; Kittleman *et al.*, 2007 ▸; Mansurova *et al.*, 2008 ▸; Osborne *et al.*, 2000 ▸). Biochemically, 5-deaza-FMN-containing Hmo or its Y128F mutant is able to oxidize (*S*)-mandelate to benzoylformate but not to benzoate, confirming that the enzyme was refolded successfully as the wild type and supporting N5 as the pivotal factor in the oxidative decarboxylation reaction [Fig. 4 ▸(*c*)]. Similarly, benzoylformate but not benzoate was found in 5-deaza-FMN_ox_-containing crystals of Hmo or its Y128F mutant soaked with (*S*)-mandelate [Figs. 4 ▸(*d*) and 4 ▸(*e*)]. No N5-acyl adducts can be found in 5-deaza-FMN-containing crystals of Hmo or its Y128F mutant soaked with benzoylformate or phenylpyruvate at various concentrations at different time intervals. Furthermore, the lack of visible electron density at the top of C5 or between C5 and C′α of benzoylformate (4.0 Å) suggests that the C4α=C5 double bond in 5-deaza-FMN is less polarizable. Our structural interrogation supports the decarboxylation of the α-ketoacid taking place after or in concert with the formation of the C′α–N5 bond. The R163L mutant (a low-activity mutant) was further examined using the nondecarboxylable substrates α-(*S*)-mandelamide [2-(*S*)-hydroxy-2-phenylethylamide] or benzoylamide (2-keto-2-phenylethylamide), in which the former is oxidized to form the latter. When the R163L mutant crystals were soaked with benzoylamide, an α-hydroxyamide-FMN adduct was formed [Fig. 4 ▸(*f*)], leading to the unequivocal conclusion that N5 of FMN_ox_ has a nucleophilic propensity and that the C′α—N5 bond is formed prior to α-ketoacid decarboxylation.

### 3.3. Four-electron oxidation to benzoate   

We propose that C4α-OOH-N5-acyl-FMN is the key intermediate in the four-electron oxidation of an α-hydroxyacid mediated by Hmo and its Y128F mutant on the basis of the following facts: (i) Hmo and its Y128F mutant catalyze the four-electron oxidation of an α-hydroxyacid via an α-ketoacid to an acid with one O atom from O_2_ incorporated into the terminal carboxylic group, (ii) H_2_O_2_ is not able to oxidize the α-ketoacid in the absence of Hmo or its Y128F mutant, (iii) the *pro-R* α-ketoacid is covalently linked to FMN_ox_ in the Y128F mutant, forming an N5-acyl-FMN_red_ adduct, (iv) the oxidation cascade stalls at the α-ketoacid using Hmo or its Y128F mutant with 5-deaza-FMN_ox_ in lieu of FMN_ox_ and (v) the *sp* ^3^ N5 in FMN_red_ is highly reactive, as exemplified in UbiX, a flavin prenyltransferase involved in bacterial ubiquinone biosynthesis (White *et al.*, 2015 ▸). The formation of the intermediate is somewhat similar to the mechanism proposed for EncM, which catalyses an oxidative Favorskii-type re­arrangement reaction (Teufel *et al.*, 2015 ▸). The major discrepancy, however, is that O_2_ in Hmo and its Y128F mutant mediates the transient formation of C4α-OOH-N5-acyl-FMN prior to its release as H_2_O_2_.

Superposition of the benzoylformate-liganded ternary complex of the Y128F mutant with that of the wild type shows no apparent discrepancies (r.m.s.d. of 0.06 Å) except for the *p*-OH group of Tyr128 (Supplementary Fig. S4). Given that the oxidative decarboxylation of an α-hydroxyacid is cata­lytically executed by the Y128F mutant, the *p*-OH group ought to play a crucial role in leverage of the oxidation cascade. This effect is commensurate with a recent report that a single mutation, C65D, of phenylacetone monooxygenase converts a monooxygenase to an oxidase (in contrast to this report) by facilitating the discharge of H_2_O_2_ (Brondani *et al.*, 2014 ▸). C4α-OOH-FMN_red_, which is a reactive intermediate in a typical monooxygenase/oxidase-catalyzed reaction, was modeled and optimized in the structure of Hmo, in which the *p*-OH group is within hydrogen-bonding distance (2.7 Å) of C4α-OOH [Fig. 3 ▸(*e*)]. On the basis of this model, the *p*-OH group is in a position to protonate C4α-OO^−^-FMN_red_ to form C4α-OOH-FMN_red_, thereby neutralizing or facilitating the discharge of H_2_O_2_ from C4α-OOH. In contrast, C4α-OO^−^-FMN_red_ may diverge in the absence of the *p*-OH group. One implication is that the Y128F mutant works like a monooxygenase, whereby Baeyer–Villiger-type reactions result. That is, the C4α-OO^−^ anion attacks the α-carbon (C′α) of *pro-R* benzoylformate to form a tetrahedral oxyanion species. Upon the collapse of the α-oxyanion the terminal carboxyl group migrates to the distal O atom of C4α-OO^−^ to form a mixed-anhydride species; subsequent hydrolysis would give rise to benzoate and formate (Torres Pazmiño *et al.*, 2010 ▸). This type of reaction, however, was ruled out because no benzoate was detected in the reactions catalyzed by the 5-deaza-FMN-containing Y128F mutant. This fact, however, underscores the importance of the *sp* ^3^ N5 of C4α-OO^−^-FMN_red_ in the four-electron oxidation reaction, where the reduced or polarized *sp* ^3^ N5 actually has a better Bürgi–Dunitz angle and is at a short distance from C′α of *pro-R* benzoylformate, favoring the formation of C4α-OO^−^-N5-alkyl-FMN_red_ before the release of H_2_O_2_.

### 3.4. Proposed catalytic mechanism of oxidative decarboxylation   

**Figure 5:**
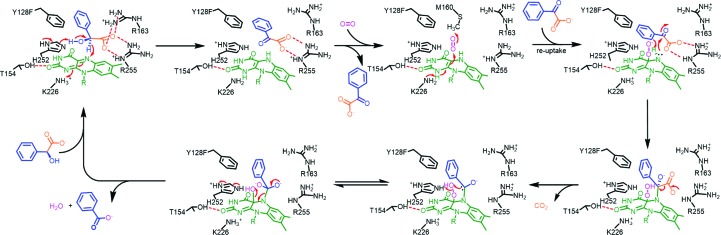
The proposed mechanisms of oxidative decarboxylation catalyzed by the Y128F mutant.

In a general four-electron oxidation reaction, one molecule of (*S*)-mandelate should theoretically yield two equivalents of H_2_O_2_. The molar ratio of H_2_O_2_ versus benzoate, however, did not follow this stoichiometry (it was much less than unity; Yeh *et al.*, 2019 ▸). This fact, in contrast, is consistent with a disproportionation reaction of FMN peroxide, in which one O atom goes to benzoate and the other ends up as water. To search for clues, we re-examined the active-site geometry of Hmo and its Y128F mutant liganded with substrates (mandelate or lactate) or products (benzoylformate or pyruvate). The redox-active center C4α=N5 of isoalloxazine is surrounded by a constellation of active-site residues [Val78 and Ala79 at the bottom and Tyr(Phe)128 and His252 at the top], where it is accessible only from the upper front side. The reaction center is sealed to form a narrow and low-dielectric milieu suitable for hydride transfer/electron tunneling when a substrate and redox-active center C4α=N5 approach each other. Interestingly, the substrate pair mandelate/benzoyl­formate fits better than the alternative pair lactate/pyruvate because of the bulky phenyl group in the former [Figs. 3 ▸(*f*) and 3 ▸(*g*)]. The redox chamber in the Y128F mutant, on the other hand, is not as tight as that in the wild type owing to the lack of the *p*-OH group [Fig. 3 ▸(*h*)]. This flaw is exacerbated when lactate (with a smaller methyl group) is used [Figs. 3 ▸(*f*) and 3 ▸(*g*)]. In this context, O_2_ is relatively accessible to FMN_red_ via a temporal space/tunnel to form C4α-OO^−^-FMN before the release of the α-ketoacid (the non-ping-pong mechanism). Meanwhile, the *pro*-*R* α-ketoacid is accessible by *sp* ^3^ N5 to form C4α-COOH-N5-oxyalkylate-FMN_red_. Upon decarboxyl­ation, a C4α-COOH-N5-aloxyl-FMN_red_ C′α carb­anion results. The C′α carbanion that intramolecularly attacks the distal O atom of C4α-OOH then leads to heterolytic cleavage of the peroxide scissile bond. Upon return of the C′α oxyanion, benzoate and H_2_O are formed in concert with the regeneration of FMN_ox_ (Fig. 5 ▸).

The peroxide anion radical:FMN semiquinone caged pair is likely to proceed through a single-electron transfer from FMN_red_ to O_2_ in a given flavoenzyme, where the reactivity depends on the active-site polarity ensemble of factors including bound water, charge distribution, hydrogen bonds, van der Waal forces *etc.* (Fagan & Palfey, 2010 ▸). The Y128C mutant (in which the bulky phenyl group is replaced by a sulfhydryl group) that can transform (*S*)-mandelate to benzoate was used to assess the extent of active-site perturbation. The structure of the Y128C mutant crystallized and soaked with (*S*)-mandelate revealed that the sulfhydryl (SH) group of the Y128C mutant has been oxidized to a sulfenyl group (S-OH), in contrast to the other sulfhydryl groups, which are not changed [Fig. 3 ▸(*i*)]. This result indicates that the sulfhydryl group of the Y128C mutant is relatively accessible and sensitive to the local unregulated reactive oxygen species (ROS; Chaiyen *et al.*, 2012 ▸).

## 4. Conclusions   

The present studies allow us to gain mechanistic insights into the reactions catalyzed by both Hmo and its Y128F mutant, in which substrate reorientation, active-site perturbation and spatiotemporal crowdedness are pivotal factors that influence the dioxygen accessibility and reaction order of the FMN_red/ox_:α-ketoacid pair in the reactions mediated by Hmo and its Y128F mutant. Given the Y128F mutation, the original reactivity of Hmo is perturbed. One stark contrast is that the electrophilic FMN_ox_ is polarizable to an ylide-like species. This species is capable of attacking an α-ketoacid to form an N5-acyl-FMN_red_ dead-end adduct, providing evidence for the first time that FMN_ox_ possesses a nucleophilic/electrophilic duality. Having confirmed the formation of the N5-acyl-FMN_red_ adduct, both the nucleophilic propensity and positional preponderance of N5 of FMN_red_ prompt us to propose that the N5-alkanol-FMN_red_ C′α carbanion is the key intermediate in the oxidative decarboxylation reaction. This intermediate reacts with dioxygen in place to form a C4α-COOH-N5-aloxyl-FMN_red_ C′α carbanion species that subsequently undergoes an intramolecular disproportionation reaction to yield benzoate and FMN_ox_, thus accounting for the ThDP/PLP/NADPH-independent oxidative decarboxylation reaction. To this end, the *p*-OH group of Tyr128 that leverages the spatial and temporal leeway over the oxidation cascade was unexpected. The α-substituent on the α-hydroxy acid that influences the accessibility of dioxygen to the reaction center is another unexpected factor. A synthetic 5-deaza-FMN_ox_ cofactor in combination with an α-hydroxyamide or α-keto­amide positively supports the proposed mechanism, in which the loose ends that benzoate is a minor product of Hmo and the major product of the Y128F mutant are tied up. An unequivocal consolidation of the proposed mechanism would be provided by the physical capture or visualization of the C4α-COOH-N5-aloxyl-FMN_red_ C′α carbanion or other relevant intermediates, which however will require future studies using advanced spectroscopic and microscopic analysis on the submicrosecond time scale using, for example, the X-ray free-electron laser technique. The present structural and biochemical elucidation nonetheless strengthens the idea that the FMN cofactor is versatile and cooperates with the active-site residues and substrates in dictating the oxidation cascade.

## Supplementary Material

PDB reference: *p*-hydroxymandelate oxidase, 5zzp

PDB reference: complex with (*S*)-mandelate, 5zzr

PDB reference: complex with benzoylformate, 6a08

PDB reference: Y128C mutant, complex with benzoylformate, 5zzz

PDB reference: Y128F mutant, 6a13

PDB reference: complex with (*S*)-mandelate, 6a0v

PDB reference: complex with 5-deazariboflavin mononucleotide, 6a1h

PDB reference: complex with 5-deazariboflavin mononucleotide and benzoic acid, 6a1l

PDB reference: complex with 5-deazariboflavin mononucleotide and benzoylformate, 6a1m

PDB reference: complex with 5-deazariboflavin mononucleotide and phenylpyruvate, 6a1p

PDB reference: complex with phenylpyruvate and riboflavin mononucleotide, 6a1r

PDB reference: complex with benzoylformate, 6a19

PDB reference: complex with malonyl–riboflavin mononucleotide, 6a21

PDB reference: complex with benzoylformate and riboflavin mononucleotide, 6a23

PDB reference: R163L mutant, complex with mandelamide–riboflavin mononucleotide, 6a3t

Chemical syntheses, supporting figures and table. DOI: 10.1107/S2059798319011938/ag5031sup1.pdf

## Figures and Tables

**Table:** Values in parentheses are for the highest resolution shell. SMA, (*S*)-mandelate; BF, benzoylformate; FMN, riboflavin mononucleotide; 5DAFMN, 5-­deazariboflavin mononucleotide; PPY, phenylpyruvate; MA_FMN, malonyl–FMN; MAAD_FMN, mandelamide–FMN.

	Hmo	Hmo–SMA	Hmo–BF	Y128F	Y128F–SMA	Y128F–BF	Y128F–5DAFMN	Y128F–5DAFMN–BA
PDB code	5zzp	5zzr	6a08	6a13	6a0v	6a19	6a1h	6a1l
Data collection								
Wavelength (Å)	1.0	1.0	1.0	1.0	1.0	1.0	1.0	1.0
Space group	*I*422	*I*422	*I*422	*I*422	*I*422	*I*422	*I*422	*I*422
*a*, *b*, *c* (Å)	137.9, 137.9, 112.3	137.7, 137.7, 111.6	137.9, 137.9, 111.8	137.5, 137.5, 112.0	137.8, 137.8, 111.8	137.9, 137.9, 112.3	137.4, 137.4, 112.4	137.7, 137.7, 111.6
α, β, γ (°)	90, 90, 90	90, 90, 90	90, 90, 90	90, 90, 90	90, 90, 90	90, 90, 90	90, 90, 90	90, 90, 90
Resolution range (Å)	30–1.39 (1.44–1.39)	30–1.31 (1.36–1.31)	30–1.55 (1.61–1.55)	30–1.70 (1.76–1.70)	30–1.39 (1.44–1.39)	30–1.55 (1.61–1.55)	30–1.36 (1.41–1.36)	30–1.40 (1.45–1.40)
*R*_merge_ † (%)	3.7 (73.0)	3.7 (67.0)	4.0 (79.0)	3.5 (69.2)	4.3 (73.1)	3.6 (75.0)	2.8 (62.0)	4.2 (81.0)
〈*I*/σ(*I*)〉	36.1 (2.4)	34.5 (2.3)	43.1 (2.9)	41.3 (3.1)	41.0 (2.9)	32.9 (2.7)	41.2 (3.4)	29.6 (2.4)
Completeness (%)	99.9 (100.0)	99.0 (100.0)	100.0 (100.0)	100.0 (100.0)	100.0 (100.0)	99.9 (100.0)	99.9 (100.0)	100.0 (100.0)
Multiplicity	9.4 (9.4)	9.8 (9.5)	12.8 (11.2)	11.2 (11.1)	12.2 (12.0)	9.6 (9.2)	9.7 (9.4)	10.5 (10.4)
Refinement								
Resolution range (Å)	30–1.39 (1.44–1.39)	30–1.31 (1.36–1.31)	30–1.55 (1.61–1.55)	30–1.70 (1.76–1.70)	30–1.39 (1.44–1.39)	30–1.55 (1.61–1.55)	30–1.36 (1.41–1.36)	30–1.40 (1.45–1.40)
*R*_work_ ‡ (%)	17.0 (28.0)	16.6 (22.9)	16.9 (22.0)	17.8 (24.2)	16.7 (22.9)	15.7 (24.4)	16.8 (23.0)	17.5 (28.0)
*R*_free_ § (%)	18.3 (30.0)	17.7 (22.3)	17.9 (20.1)	20.0 (26.2)	18.4 (23.1)	17.3 (26.6)	18.1 (26.0)	18.6 (30.1)
R.m.s. deviations								
Bond lengths (Å)	0.010	0.009	0.008	0.018	0.008	0.009	0.017	0.006
Bond angles (°)	1.41	1.41	1.26	1.490	1.3361	1.460	1.421	0.982
No. of reflections	100548	112497	75720	58599	98014	74156	114232	104841
No. of atoms								
Protein	2785	2622	2684	2510	2636	2703	2548	2574
Ligand/ion	31	53	65	31	53	53	31	55
Water	403	402	329	316	400	400	440	335
*B* factors (Å^2^)								
Protein	18.2	19.7	19.1	18.6	16.9	21.9	18.4	20.5
Ligand/ion	10.7	19.0	20.6	10.8	17.8	29.4	12.1	17.1
Water	32.4	33.9	31.7	32.1	32.2	37.3	34.9	26.6

**Table:** 

	Y128F–5DAFMN–BF	Y128F–5DAFMN–PPY	Y128F–PPY–FMN	Y128F–MA_FMN	Y128F–BF–FMN	Y128C–BF	R163L–MAAD_FMN
PDB code	6a1m	6a1p	6a1r	6a21	6a23	5zzz	6a3t
Data collection							
Wavelength (Å)	1.0	1.0	1.0	1.0	1.0	1.0	1.0
Space group	*I*422	**I*422*	*I*422	*I*422	*I*422	*I*422	*I*422
*a*, *b*, *c* (Å)	137.9, 137.9, 111.2	138.0, 138.0, 111.1	138.1, 138.1, 112.1	137.6, 137.6, 112.1	137.8, 137.8, 111.8	138.2, 138.2, 112.1	137.4, 137.4, 111.7
α, β, γ (°)	90, 90, 90	90, 90, 90	90, 90, 90	90, 90, 90	90, 90, 90	90, 90, 90	90, 90, 90
Resolution range (Å)	30–1.55 (1.61–1.55)	30–1.51 (1.56–1.51)	30–1.65 (1.71–1.65)	30–1.50 (1.55–1.50)	30–1.65 (1.71–1.65)	30–1.45 (1.50–1.45)	30–2.51 (2.60–2.51)
*R*_merge_ † (%)	3.9 (57.0)	3.6 (72.1)	4.4 (73.0)	3.2 (64.3)	3.8 (70.1)	4.4 (69.2)	10.1 (66.0)
〈*I*/σ(*I*)〉	40.8 (2.4)	30.4 (2.3)	36.8 (3.16)	40.0 (3.2)	28.4 (2.0)	33.5 (2.6)	15.2 (2.4)
Completeness (%)	99.7 (97.2)	99.8 (98.3)	100.0 (100.0)	99.9 (100.0)	99.3 (100.0)	99.9 (100.0)	99.9 (100.0)
Multiplicity	11.4 (9.1)	9.4 (7.7)	12.2 (12.1)	9.8 (9.8)	9.1 (8.8)	9.9 (9.9)	9.5 (9.3)
Refinement							
Resolution range (Å)	30–1.55 (1.61–1.55)	30–1.51 (1.56–1.51)	30–1.65 (1.71–1.65)	30–1.50 (1.55–1.50)	30–1.65 (1.71–1.65)	30–1.45 (1.50–1.45)	30–2.51 (2.60–2.51)
*R*_work_ ‡ (%)	17.8 (25.7)	17.0 (26.1)	16.3 (20.6)	16.7 (22.4)	17.3 (24.1)	17.5 (22.8)	17.8 (21.6)
*R*_free_ § (%)	20.1 (26.5)	19.7 (26.8)	18.5 (22.5)	19.0 (22.9)	19.4 (24.9)	19.2 (27.2)	22.4 (29.1)
R.m.s. deviations							
Bond lengths (Å)	0.018	0.015	0.019	0.018	0.006	0.010	0.008
Bond angles (°)	1.501	1.357	1.529	1.557	1.173	1.300	1.29
No. of reflections	75042	83439	64602	84212	59812	93717	17837
No. of atoms							
Protein	2485	2539	2586	2626	2606	2481	2496
Ligand/ion	53	44	41	37	51	42	43
Water	298	331	303	375	301	357	91
*B* factors (Å^2^)							
Protein	19.3	22.2	17.9	19.0	19.1	18.7	40.29
Ligand/ion	14.8	18.2	11.7	16.7	16.6	20.0	40.89
Water	34.2	35.2	30.2	33.2	30.4	31.6	38.94

† *R*_merge_ = 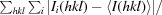 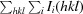, where 〈*I*(*hkl*)〉 is the average intensity value of the equivalent reflections.

‡ *R*_work_ = 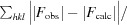 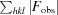.

§ *R*_free_ was calculated from 5% of data that were randomly excluded from refinement.

## References

[bb1] Afonine, P. V., Grosse-Kunstleve, R. W., Echols, N., Headd, J. J., Moriarty, N. W., Mustyakimov, M., Terwilliger, T. C., Urzhumtsev, A., Zwart, P. H. & Adams, P. D. (2012). *Acta Cryst.* D**68**, 352–367. 10.1107/S0907444912001308 PMC3322595 22505256

[bb2] Baron, R., McCammon, J. A. & Mattevi, A. (2009). *Curr. Opin. Struct. Biol.* **19**, 672–679. 10.1016/j.sbi.2009.10.003 19896366

[bb3] Baron, R., Riley, C., Chenprakhon, P., Thotsaporn, K., Winter, R. T., Alfieri, A., Forneris, F., van Berkel, W. J., Chaiyen, P., Fraaije, M. W., Mattevi, A. & McCammon, J. A. (2009). *Proc. Natl Acad. Sci. USA*, **106**, 10603–10608. 10.1073/pnas.0903809106 PMC2698890 19541622

[bb4] Brondani, P. B., Dudek, H. M., Martinoli, C., Mattevi, A. & Fraaije, M. W. (2014). *J. Am. Chem. Soc.* **136**, 16966–16969. 10.1021/ja508265b 25423359

[bb5] Carlson, E. E. & Kiessling, L. L. (2004). *J. Org. Chem.* **69**, 2614–2617. 10.1021/jo049859f 15049673

[bb6] Chaiyen, P., Fraaije, M. W. & Mattevi, A. (2012). *Trends Biochem. Sci.* **37**, 373–380. 10.1016/j.tibs.2012.06.005 22819837

[bb41] Chen, Z.-W., Vignaud, C., Jaafar, A., Levy, B., Gueritte, F., Guenard, D., Lederer, F. & Mathews, F. S. (2012). *Biochimie*, **94**, 1172–1179. 10.1016/j.biochi.2012.02.003 22342614

[bb7] Choong, Y. S. & Massey, V. (1980). *J. Biol. Chem.* **255**, 8672–8677. 7410388

[bb8] Dai, X., Mashiguchi, K., Chen, Q. G., Kasahara, H., Kamiya, Y., Ojha, S., DuBois, J., Ballou, D. & Zhao, Y. (2013). *J. Biol. Chem.* **288**, 1448–1457. 10.1074/jbc.M112.424077 PMC3548458 23188833

[bb9] DeLano, W. L. (2002). *PyMOL*. http://www.pymol.org.

[bb10] Emsley, P., Lohkamp, B., Scott, W. G. & Cowtan, K. (2010). *Acta Cryst.* D**66**, 486–501. 10.1107/S0907444910007493 PMC2852313 20383002

[bb11] Fagan, R. L. & Palfey, B. A. (2010). *Comprehensive Natural Products II: Chemistry and Biology*, edited by H.-W. Liu & L. Mander, Vol. 7, pp. 37–113. Kidlington: Elsevier.

[bb12] Ghisla, S. & Massey, V. (1977). *J. Biol. Chem.* **252**, 6729–6735. 19476

[bb13] Ghisla, S. & Massey, V. (1989). *Eur. J. Biochem.* **181**, 1–17. 10.1111/j.1432-1033.1989.tb14688.x 2653819

[bb14] Ghisla, S., Massey, V. & Choong, Y. S. (1979). *J. Biol. Chem.* **254**, 10662–10669. 500603

[bb15] Giegel, D. A., Williams, C. H. & Massey, V. (1990). *J. Biol. Chem.* **265**, 6626–6632. 2324094

[bb16] Hefti, M. H., Milder, F. J., Boeren, S., Vervoort, J. & van Berkel, W. J. (2003). *Biochim. Biophys. Acta*, **1619**, 139–143. 10.1016/s0304-4165(02)00474-9 12527109

[bb17] Heroux, A., Bozinovski, D. M., Valley, M. P., Fitzpatrick, P. F. & Orville, A. M. (2009). *Biochemistry*, **48**, 3407–3416. 10.1021/bi8023042 PMC2754721 19265437

[bb18] Kittleman, W., Thibodeaux, C. J., Liu, Y.-N., Zhang, H. & Liu, H.-W. (2007). *Biochemistry*, **46**, 8401–8413. 10.1021/bi700286a PMC2515275 17585782

[bb19] Lockridge, O., Massey, V. & Sullivan, P. A. (1972). *J. Biol. Chem.* **247**, 8097–8106. 4640938

[bb20] Lopalco, A., Dalwadi, G., Niu, S., Schowen, R. L., Douglas, J. & Stella, V. J. (2016). *J. Pharm. Sci.* **105**, 705–713. 10.1002/jps.24653 PMC4814373 26422524

[bb21] Mansurova, M., Koay, M. S. & Gärtner, W. (2008). *Eur. J. Org. Chem.* **2008**, 5401–5406.

[bb22] McCoy, A. J., Grosse-Kunstleve, R. W., Adams, P. D., Winn, M. D., Storoni, L. C. & Read, R. J. (2007). *J. Appl. Cryst.* **40**, 658–674. 10.1107/S0021889807021206 PMC2483472 19461840

[bb23] Milczek, E. M., Bonivento, D., Binda, C., Mattevi, A., McDonald, I. A. & Edmondson, D. E. (2008). *J. Med. Chem.* **51**, 8019–8026. 10.1021/jm8011867 PMC2706497 19053775

[bb50] Murshudov, G. N., Skubák, P., Lebedev, A. A., Pannu, N. S., Steiner, R. A., Nicholls, R. A., Winn, M. D., Long, F. & Vagin, A. A. (2011). *Acta Cryst.* D**67**, 355–367. 10.1107/S0907444911001314 PMC3069751 21460454

[bb24] Osborne, A., Thorneley, R. N., Abell, C. & Bornemann, S. (2000). *J. Biol. Chem.* **275**, 35825–35830. 10.1074/jbc.M005796200 10956653

[bb25] Otwinowski, Z. & Minor, W. (1997). *Methods Enzymol.* **276**, 307–326. 10.1016/S0076-6879(97)76066-X 27754618

[bb26] Razeto, A., Mattiroli, F., Carpanelli, E., Aliverti, A., Pandini, V., Coda, A. & Mattevi, A. (2007). *Structure*, **15**, 683–692. 10.1016/j.str.2007.04.009 17562315

[bb27] Stepanova, A. N., Yun, J., Robles, L. M., Novak, O., He, W., Guo, H., Ljung, K. & Alonso, J. M. (2011). *Plant Cell*, **23**, 3961–3973. 10.1105/tpc.111.088047 PMC3246335 22108406

[bb28] Sucharitakul, J., Phongsak, T., Entsch, B., Svasti, J., Chaiyen, P. & Ballou, D. P. (2007). *Biochemistry*, **46**, 8611–8623. 10.1021/bi7006614 17595116

[bb29] Teufel, R., Stull, F., Meehan, M. J., Michaudel, Q., Dorrestein, P. C., Palfey, B. & Moore, B. S. (2015). *J. Am. Chem. Soc.* **137**, 8078–8085. 10.1021/jacs.5b03983 PMC4720136 26067765

[bb30] Thotsaporn, K., Chenprakhon, P., Sucharitakul, J., Mattevi, A. & Chaiyen, P. (2011). *J. Biol. Chem.* **286**, 28170–28180. 10.1074/jbc.M111.241836 PMC3151062 21680741

[bb31] Torres Pazmiño, D. E., Dudek, H. M. & Fraaije, M. W. (2010). *Curr. Opin. Chem. Biol.* **14**, 138–144. 10.1016/j.cbpa.2009.11.017 20015679

[bb33] Walsh, C. (1980). *Mol. Biol. Biochem. Biophys.* **32**, 62–77. 10.1007/978-3-642-81503-4_5 7442653

[bb34] Walsh, C., Lockridge, O., Massey, V. & Abeles, R. (1973). *J. Biol. Chem.* **248**, 7049–7054. 4147556

[bb35] Walsh, C. T. (1984). *Annu. Rev. Biochem.* **53**, 493–535. 10.1146/annurev.bi.53.070184.002425 6433782

[bb36] Walsh, C. T. & Wencewicz, T. A. (2013). *Nat. Prod. Rep.* **30**, 175–200. 10.1039/c2np20069d PMC3518583 23051833

[bb37] White, M. D., Payne, K. A. P., Fisher, K., Marshall, S. A., Parker, D., Rattray, N. J. W., Trivedi, D. K., Goodacre, R., Rigby, S. E. J., Scrutton, N. S., Hay, S. & Leys, D. (2015). *Nature (London)*, **522**, 502–506. 10.1038/nature14559 PMC4988493 26083743

[bb40] Winn, M. D., Ballard, C. C., Cowtan, K. D., Dodson, E. J., Emsley, P., Evans, P. R., Keegan, R. M., Krissinel, E. B., Leslie, A. G. W., McCoy, A., McNicholas, S. J., Murshudov, G. N., Pannu, N. S., Potterton, E. A., Powell, H. R., Read, R. J., Vagin, A. & Wilson, K. S. (2011). *Acta Cryst.* D**67**, 235–242. 10.1107/S0907444910045749 PMC3069738 21460441

[bb38] Wu, T., Ling, K.-Q., Sayre, L. M. & McIntire, W. S. (2005). *Biochem. Biophys. Res. Commun.* **326**, 483–490. 10.1016/j.bbrc.2004.11.054 15582603

[bb39] Yeh, H.-W., Lin, K.-H., Lyu, S.-Y., Li, Y.-S., Huang, C.-M., Wang, Y.-L., Shih, H.-W., Hsu, N.-S., Wu, C.-J. & Li, T.-L. (2019). *Acta Cryst.* D**75**, 733–742. 10.1107/S2059798319009574 PMC6677016 31373572

