# Supplementary material for: The flavin mononucleotide cofactor in α-hydroxyacid oxidases exerts its electrophilic/nucleophilic duality in control of the substrate-oxidation level
Source: Acta Crystallogr D Struct Biol. 2019 Sep 24;75(Pt 10):918–29. doi: 10.1107/S2059798319011938 (PMC6778850; doi:10.1107/S2059798319011938)
Supplement: Supplementary file 1 [file d-75-00918-sup1.pdf]

STRUCTURAL
BIOLOGY

Volume 75 (2019)

Supporting information for article:

The flavin mononucleotide cofactor in α-hydroxyacid oxidases exerts its electrophilic/nucleophilic duality in control of the substrate oxidation level

Syue-Yi Lyu, Kuan-Hung Lin, Hsien-Wei Yeh, Yi-Shan Li, Chun-Man Huang, Yung-Lin Wang, Hao-Wei Shih, Ning-Shian Hsu, Chang-Jer Wu and Tsung-Lin Li

S1. Materials and Methods

S1.1. Chemical synthesis and characterization

Each chemically synthesized compounds was purified using column chromatography and characterized by MS or NMR for enzymatic experiments unless otherwise stated. ^{1}H and ^{13}C NMR spectra in DMSO-d_6 or CD_3OD for selected compounds were recorded on Bruker Avance 600 spectrometers equipped with CryoProbe™. ^{1}H NMR spectra were reported in units of ppm relative to tetramethylsilane (TMS). Peak information is presented in the following order: chemical shift (δ), peak multiplicity (*s* = singlet, *d* = doublet, *t* = triplet, *m* = multiplet, *dd* = doublet of doublet), coupling constant (*J*) and proton number. NMR peaks were presented as follows:

S1.2. Synthesis of 5-deazaflavin mononucleotide

To obtain the 5-deazaflavin mononucleotide (7), commercially available compounds 3,4-dimethylaniline (1), $NaCNBH_3$ and D-ribose were used as starting materials following the synthetic procedure described in Scheme S1.

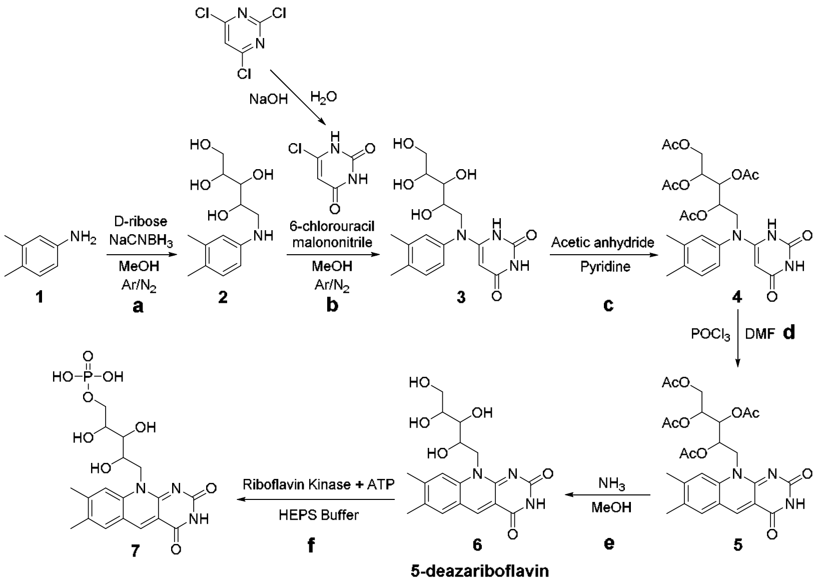

Scheme S1.

S1.2.1. Synthesis of 3,4-dimethyl-N(ribityl)-aniline (2)

3,4-Dimethylaniline (3 g, 25 mmol), $NaCNBH_3$ (3.15 g, 50 mmol) and D-ribose (7.5 g, 50 mmol) were added in anhydrous MeOH (100 mL). A mixture of reactants was heated at 40°C and reflux for 48 hrs in an anaerobic condition. The reaction solvent was evaporated and then dissolved in 1 M HCl (20 mL) with stirring until gas evolution ceased. A saturated $NaHCO_3$ solution was added to the mixture with pH at 7.0, then the mixture was extracted with EtOAc (4 × 40 mL). The combined organic layers were washed with water (2 × 25 mL) and dried. Removal of the solvent afforded a white powder 3,4-dimethyl-N(ribityl)-aniline (**2**, 4.1 g, 65 %). LC-MS: m/z 278.36 $[M+Na]^+$.

S1.2.2. Synthesis of bicyclic intermediate (3)

3,4-Dimethyl-N(ribityl)-aniline (**2**, 3.1 g, 12.0 mmol), 6-chlorouracil (1.06 g, 7.2 mmol) and

malononitrile (480 mg, 7.2 mmol) were added in anhydrous MeOH (100 mL). A mixture of reactants was heated at 40°C and reflux for 48 hrs in an anaerobic condition. Removal of solvent afforded a brown oil of bicyclic intermediate (**3**). LC-MS: m/z 366.40 $[M+H]^+$.

S1.2.3. Synthesis of acetylated bicyclic intermediate (4)

The unpurified bicyclic intermediate (**4**, 3 g) was dissolved in pyridine (25 mL) with acetic anhydride (3 mL, 31.7 mmol). The reaction mixture was stirred vigorously at room temperature for 16 hrs. After condensing the reaction mixture with rotary evaporator, the remaining was re-dissolved in $CHCl_2$ and washed by water and brine. The crude residue was purified by column chromatography ($CHCl_3$:MeOH = 10:1 (v/v)) to afford 1 g of the compound (an orange oil). LC-MS: m/z 534.60 $[M+H]^+$.

S1.2.4. Synthesis of 5-deazariboflavin tetraacetate (5)

The acetylated bicyclic intermediate (**4**, 2 g, 3.75 mmol) was dissolved in DMF (15 mL), and $POCl_3$ (1.5 mL, 2.47 g, 15.8 mmol) was added dropwise. The reaction was allowed to warm to room temperature and stirred for 1 hr, then heated to 100°C for 15 mins. The aqueous phase was extracted with $CHCl_2$ (3 × 30 mL) and washed with water (2 × 20 mL). The organic layers were combined, washed, dried, filtered, and evaporated under reduced pressure to afford 5-deazariboflavin tetraacetate **5**. LC-MS: m/z 544.70 $[M+H]^+$.

S1.2.5. Synthesis of 5-deazariboflavin (6)

Crude 5-deazariboflavin tetraacetate (**5**, 1 g, 1.84 mmol) was dissolved in methanolic ammonia

(25 mL) and stirred at room temperature for 12 hours. The solution was evaporated under reduced pressure to yield a brilliant yellow oil. The crude residue was further purified by column chromatography ($CHCl_3$:MeOH = 20:1 (v/v), 60% overall yield). LC-MS: m/z 376.56 $[M+H]^+$. ^{1}H-NMR of 5-deazariboflavin (600 MHz, DMSO-d6): δ 2.34 (*s*, 3H), 2.45 (*s*, 3H), 3.64 (*m*, 2H), 7.88 (*s*, 1H), 7.95 (*s*, 1H), 8.87 (*s*, 1H), 11.1 (*s*, NH); ^{13}C NMR of 5-deazariboflavin (600 MHz, DMSO-d6): δ18.8 (CH_3), 21.1 (CH_3), 47.4 (C1'), 63.5 (C5'), 69.8 (C2'), 73.0 (C3'), 73.8 (C4'), 113.9 (C4α), 118.1 (C6α), 119.9 (C9), 130.1 (C8), 133.9 (C6), 140.1 (C7), 141.4 (C5), 146.3 (C9α), 156.7 (C2), 157.7 (C10α), 162.4 (C4) (Fig. S5).

A.

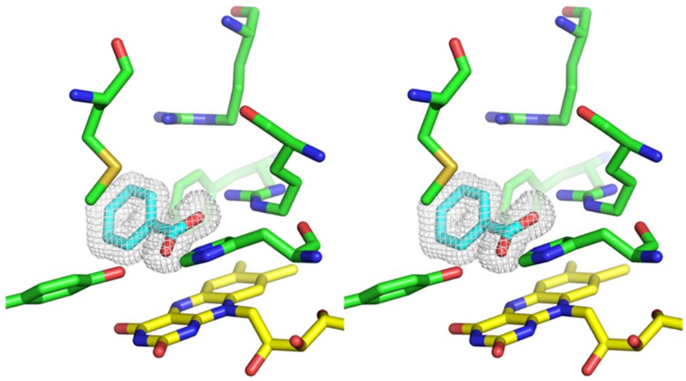

B.

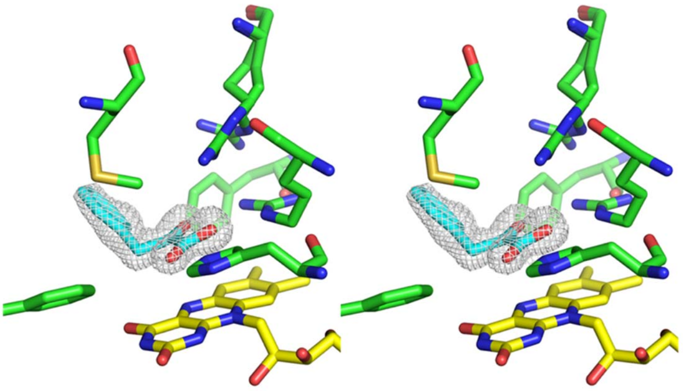

C.

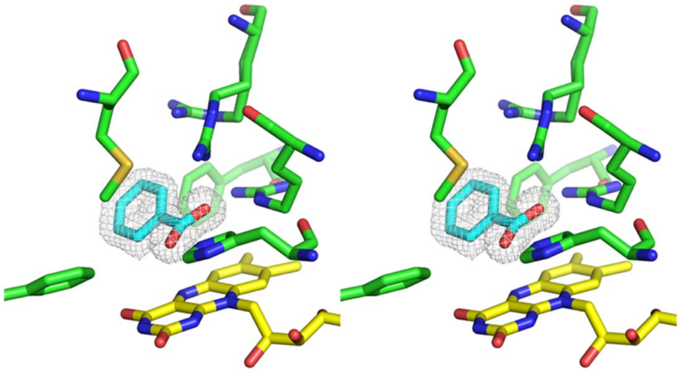

D.

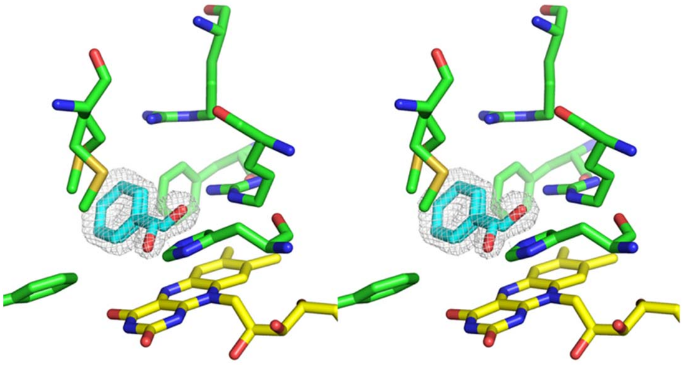

E.

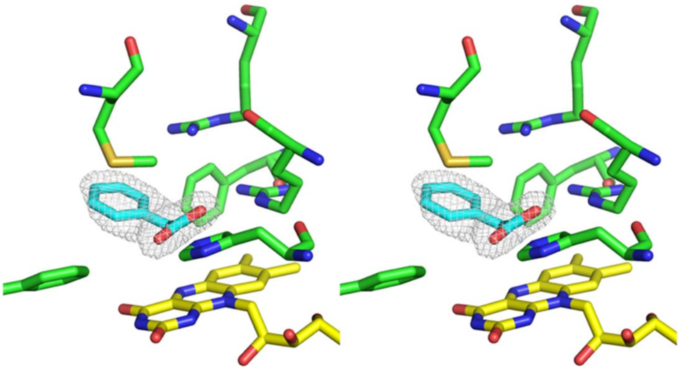

F.

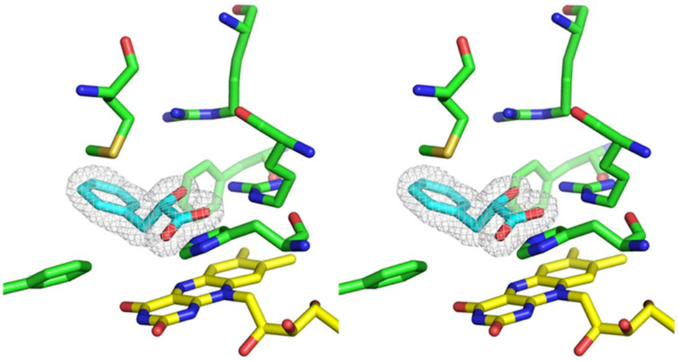

G.

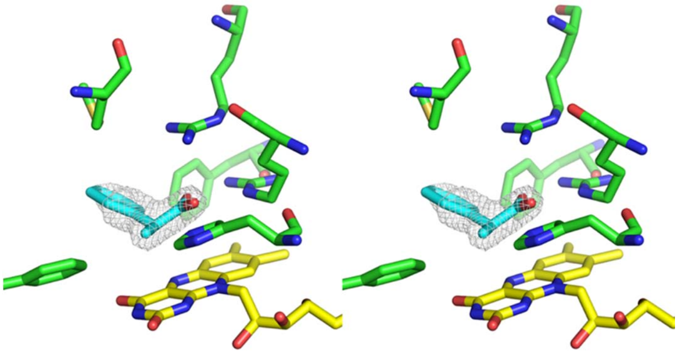

H.

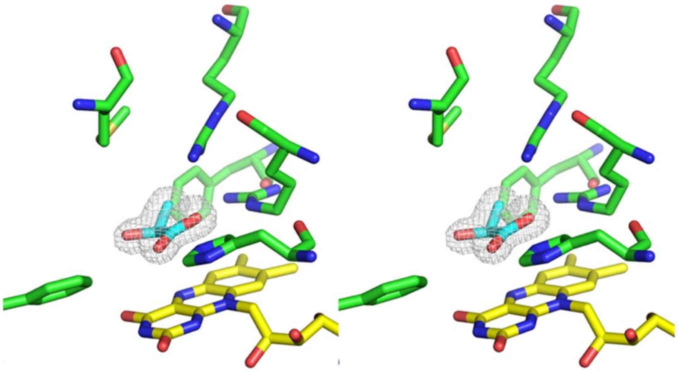

I.

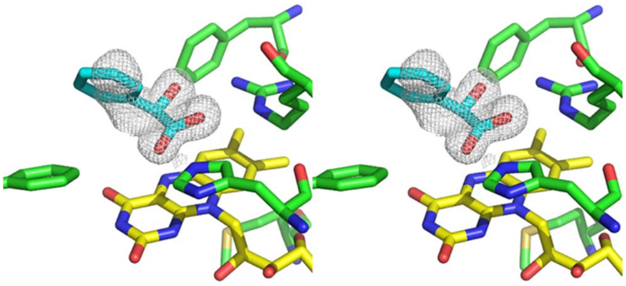

J.

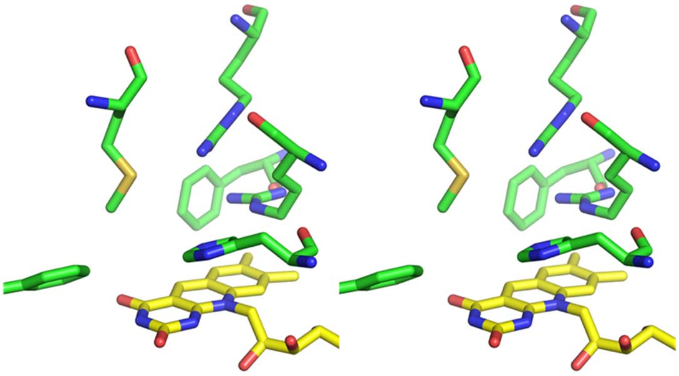

K.

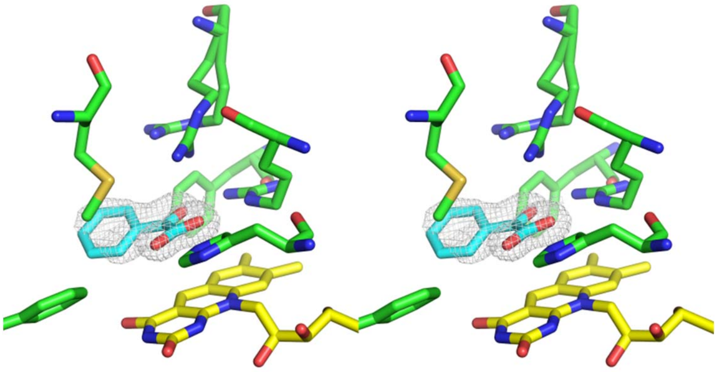

L.

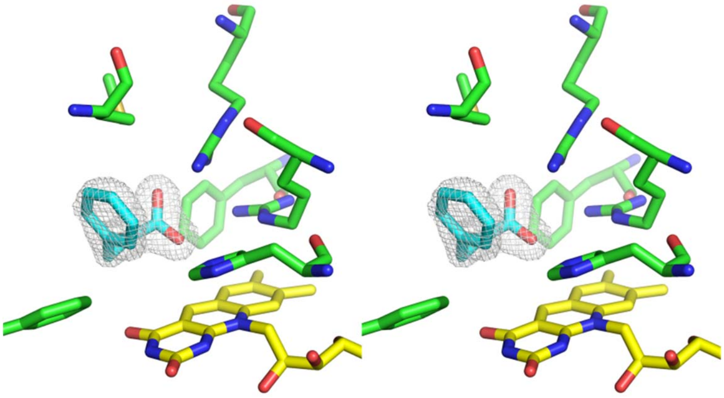

M.

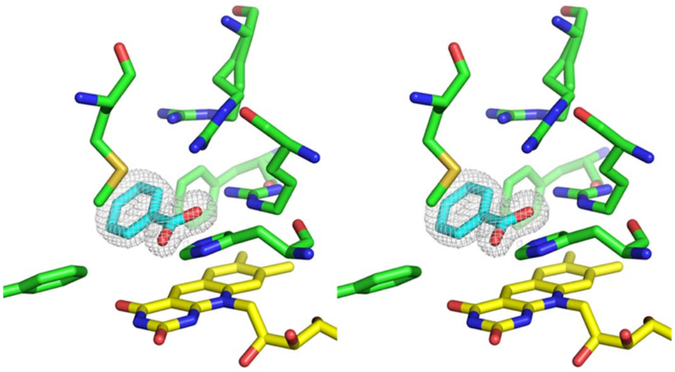

Figure S1 The stereoview of ternary complexes of Hmo/Y128F**.** The crystal structure of Hmo in complex with benzoate (A). The crystal structure of Y128F in complex with phenylpyruvate (B), benzoate (C), benzaldehyde (D), 2-phenylacetate (E), 2-hydroxy-3-phenylpropanoate (F), 2-phenylpropanoate (G), 2-hydroxypropanoate (H), or (*R*)-mandelate (I). The binary complex of Y128F harboring 5-deazariboflavin mononucleotide (J). The ternary complex of Y128F harboring 5-deazariboflavin mononucleotide and benzoylformate (K). The ternary complex of Y128F harboring 5-deazariboflavin mononucleotide and phenylpyruvate (L). The ternary complex of Y128F harboring 5-deazariboflavin mononucleotide and benzoate (M). The $2F_o$-F_c electron density maps are contoured at 1 σ in grey.

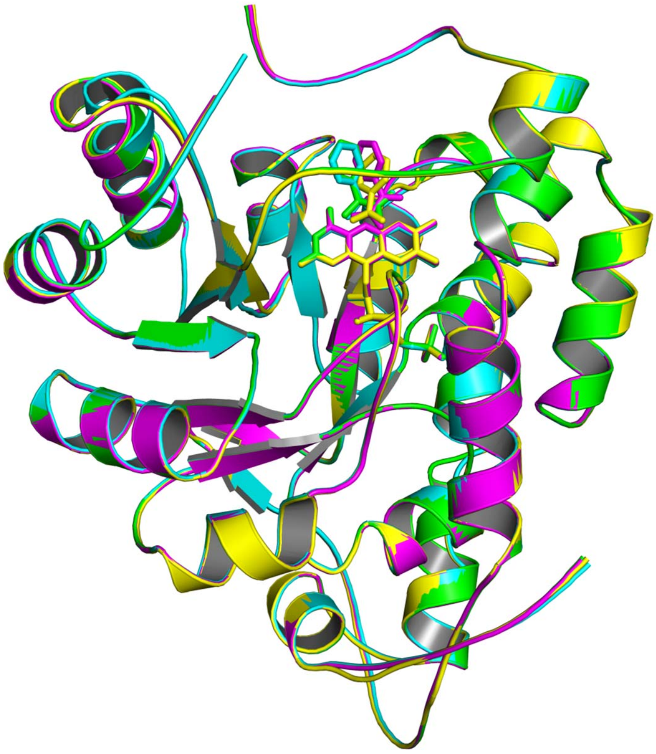

Figure S2 Superposition of ternary complexes of Y128F. Superposition of the ternary Hmo/Y128F complexes containing (*S*)-mandelate, benzoylformate or benzoate shows low average root-mean-square deviations (rmsd) with 0.07, 0.06, and 0.07 Å, respectively, for Cα backbone atoms. Hmo complexed with (*S*)-mandelate, and Y128F complexed with (*S*)-mandelate, benzoylformate, or benzoate are colored cyan, green, magentas or yellow, respectively.

A.

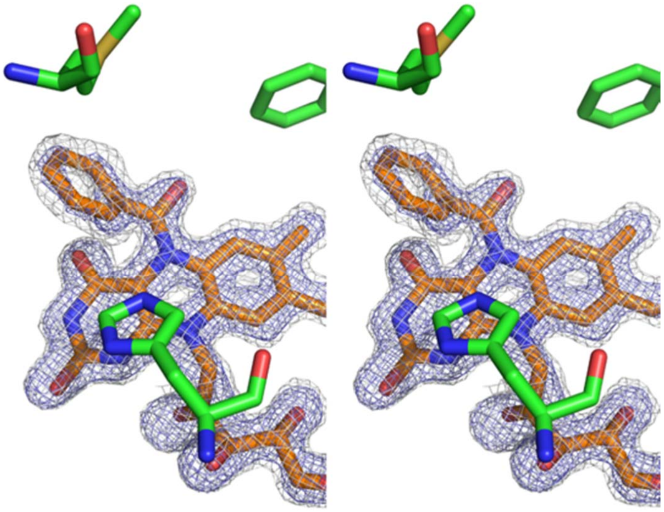

B.

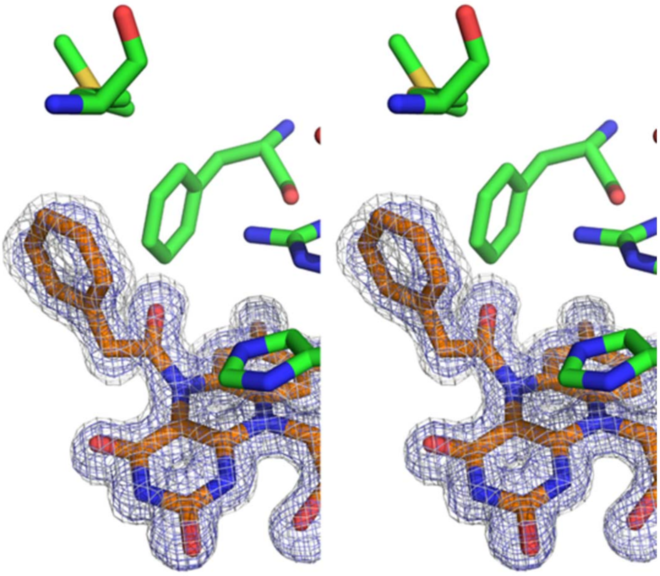

C.

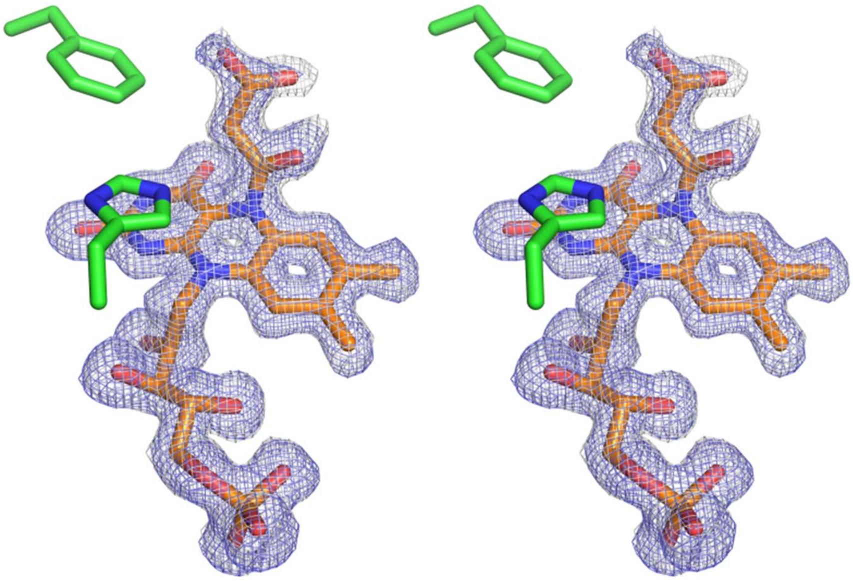

Figure S3 The stereoview of N5-adducts in Y128F crystal complexes. (A) The N5-benzyl-FMN adduct in Y128F. (B) The N5-phenylacetyl-FMN adduct in Y128F. (C) The N5-malonyl-FMN adduct in Y128F. The $2F_o$-F_c electron density maps are contoured at 1 σ in grey; the simulated annealing omit maps are contoured at 5 σ in blue.

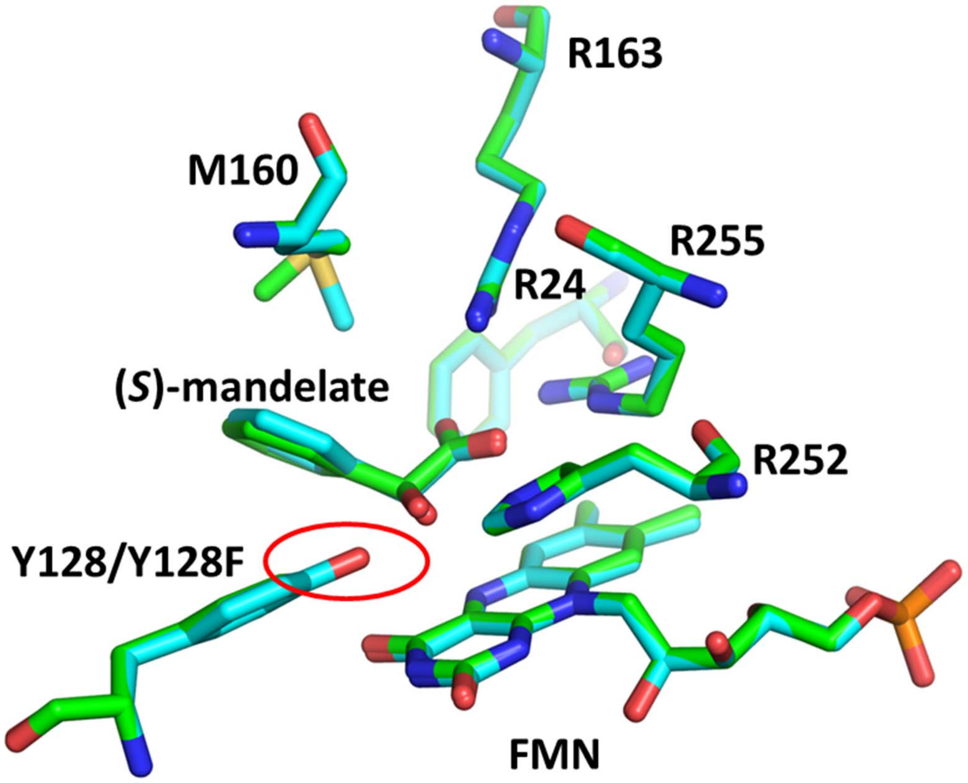

Figure S4 Superposition of ternary complexes of Hmo versus Y128F. Except the *p*-OH at residue Y128 (highlighted), superposition of the benzoylformate-containing ternary complex of Y128F versus that of WT shows no apparent differences for both the ligands and proteins. The complexes of Hmo and Y128F are colored cyan and green, respectively.

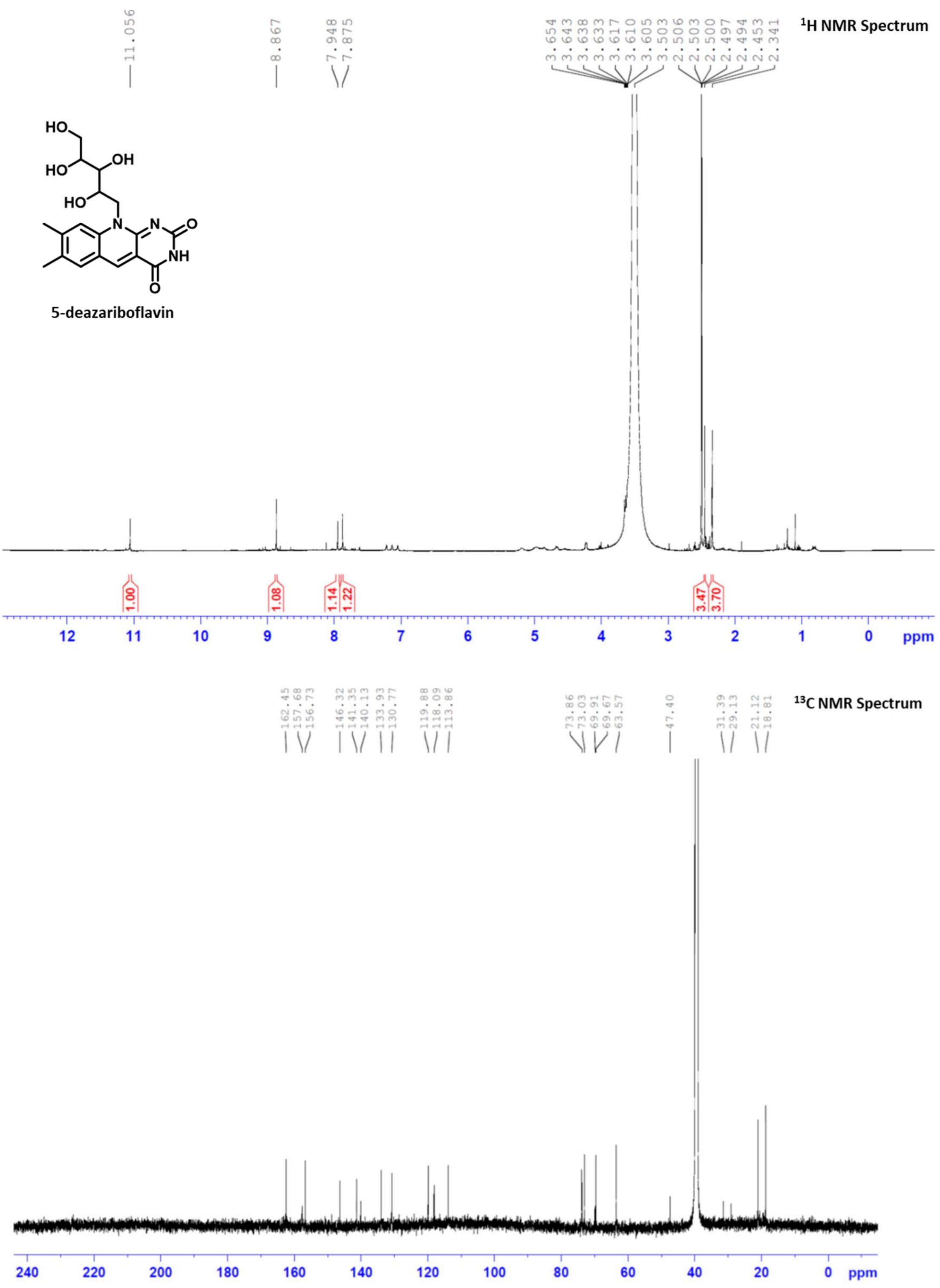

Figure S5 NMR analyses for 5-deazariboflavin. NMR spectra include ^{1}H (top panel), and ^{13}C (bottom panel).

Table S1 Primers used for preparing Hmo mutants

**Y128C**	
Forward	5'-CATCCCGCAGCC**AGC**ACAGCTGGAACCAC-3'
Reverse	5'-GTGGTTCCAGCTGT**GCT**GGCTGCGGGATG-3'
**Y128F**	
Forward	5'-CCGTGGTTCCAGCTGTTCTGGCTGCGGGATGA-3'
Reverse	5'-TCATCCCGCAGCCAGAACAGCTGGAACCACGG-3'
**R163L**	
Forward	5'-CCGTGGTTCCAGCTGTTCTGGCTGCGGGATGA-3'
Reverse	5'-TCATCCCGCAGCCAGAACAGCTGGAACCACGG-3'
